# *Bemisia tabaci* on Vegetables in the Southern United States: Incidence, Impact, and Management

**DOI:** 10.3390/insects12030198

**Published:** 2021-02-26

**Authors:** Yinping Li, George N. Mbata, Somashekhar Punnuri, Alvin M. Simmons, David I. Shapiro-Ilan

**Affiliations:** 1 Agricultural Research Station, Fort Valley State University, 1005 State University Drive, Fort Valley, GA 31030, USA; yinping.li@fvsu.edu (Y.L.); Punnuris@fvsu.edu (S.P.); 2 U.S. Vegetable Laboratory, USDA-ARS, 2700 Savannah Highway, Charleston, SC 29414, USA; alvin.simmons@usda.gov; 3 SE Fruit and Tree Nut Research Unit, USDA-ARS, 21 Dunbar Road, Byron, GA 31008, USA; david.shapiro@usda.gov

**Keywords:** whiteflies, identification, biology, plant damage, IPM

## Abstract

**Simple Summary:**

The sweetpotato whitefly, *Bemisia tabaci*, was initially discovered in the United States in 1894 but was not considered an economic insect pest on various agricultural crops across the southern and western states. After the introduction of *B. tabaci* Middle East-Asia Minor 1 (MEAM1) into the United States around 1985, the insect rapidly spread throughout the Southern United States to Texas, Arizona, and California. Extreme field outbreaks occurred on vegetable and other crops in those areas. The sweetpotato whitefly is now regarded as one of the most destructive insect pests in vegetable production systems in the Southern United States. The direct and indirect plant damage caused by *B. tabaci* has led to substantial economic losses in vegetable crops. *Bemisia tabaci* outbreaks on vegetables in Georgia resulted in significant economic losses of 132.3 and 161.2 million US dollars (USD) in 2016 and 2017, respectively. Therefore, integrated pest management (IPM) tactics are warranted, including cultural control by manipulation of production practices, resistant vegetable varieties, biological control using various natural enemies, and the judicious use of insecticides.

**Abstract:**

*Bemisia tabaci* Gennadius (Hemiptera: Aleyrodidae) is among the most economically important insect pests of various vegetable crops in the Southern United States. This insect is considered a complex of at least 40 morphologically indistinguishable cryptic species. *Bemisia tabaci* Middle East-Asia Minor 1 (MEAM1) was initially introduced in the United States around 1985 and has since rapidly spread across the Southern United States to Texas, Arizona, and California, where extreme field outbreaks have occurred on vegetable and other crops. This pest creates extensive plant damage through direct feeding on vegetables, secreting honeydew, causing plant physiological disorders, and vectoring plant viruses. The direct and indirect plant damage in vegetable crops has resulted in enormous economic losses in the Southern United States, especially in Florida, Georgia, and Texas. Effective management of *B. tabaci* on vegetables relies mainly on the utilization of chemical insecticides, particularly neonicotinoids. However, *B. tabaci* has developed considerable resistance to most insecticides. Therefore, alternative integrated pest management (IPM) strategies are required, such as cultural control by manipulation of production practices, resistant vegetable varieties, and biological control using a suite of natural enemies for the management of the pest.

## 1. Introduction

Whiteflies (Hemiptera: Aleyrodidae) have long been known as an economically important insect pest worldwide [1,2]. There are over 1550 species of whiteflies on a global scale [3,4]. The most widely known species of whiteflies are the sweetpotato whitefly, *Bemisia tabaci* Gennadius (Hemiptera: Aleyrodidae), and the greenhouse whitefly, *Trialeurodes vaporariorum* Westwood (Hemiptera: Aleyrodidae) [5]. These two species are among the most destructive insect pests of agricultural crops, vegetables, and ornamental plants in the Southern United States (e.g., Florida, Georgia, North Carolina, South Carolina, Louisiana, Tennessee, and Texas) [6]. Specifically, *B. tabaci* is a major threat to vegetable production in the Southern United States [7,8,9]. The introduction of *B. tabaci* Middle East-Asia Minor 1 (MEAM1) into the United States around 1985 [10,11,12] resulted in substantial economic losses due to direct and indirect damage on vegetable crops [13,14,15]. Therefore, in integrated pest management (IPM) program of *B. tabaci*, multiple complementary tactics are warranted, including cultural measures, host plant resistance, biological control, along with the judicious use of chemical insecticides. This review summarizes the incidence, impact, and management of *B. tabaci* on vegetables in the Southern United States, as well as general information on species identification, biology, and plant damage.

## 2. Identification

The family Aleyrodidae in the insect order Hemiptera comprises insects that derive their common name “whiteflies” from the mealy white wax covering on the wings and bodies of adults [6]. Among the 1556 species of whiteflies in 161 genera around the world [3,4], only about 150 (10%) of these occur in the United States [16], and about 76 out of 150 species are known to occur in the Southeastern United States [6,17]. Of these 76 species of whiteflies, 33 species from 20 genera are considered to be common and economically important [6].

The three main species that cause problems in vegetables in North America and Hawaii are *B. tabaci*, *T. vaporariorum*, and the bandedwinged whitefly [*Trialeurodes abutiloneus* Haldeman (Hemiptera: Aleyrodidae)] [18]. *Bemisia tabaci* is a primary insect pest of vegetable crops throughout the Southern United States [7,8,9].

*Bemisia tabaci* was initially documented by Gennadius as *Aleurodes tabaci*, a whitefly found in Greece [19]. Extensive studies on the nomenclature have demonstrated that this entity is a complex of cryptic species (formerly called biotypes) with considerable genetic diversity [20,21,22,23,24]. Body morphology (e.g., setae and spines of nymphs, and adult body lengths) among *B. tabaci* cryptic species is the same; however, they can differ in their biological characteristics (e.g., ability to induce phytotoxic responses, plant virus transmission capabilities, pesticide resistance expression, symbiotic bacteria, and host plants), biochemical attributes (e.g., diagnostic esterase banding pattern), and mitochondrial COI (cytochrome *c* oxidase subunit 1) DNA sequence [25,26,27,28,29,30]. Confirmation of a given cryptic species is determined by DNA analysis. The *B. tabaci* MEAM1 (formally called biotype B) group is the most common around the world, which possibly originated from the Middle East Asia Minor region [31]. *Bemisia tabaci* MEAM1 was identified in the late 1980s [26]. The field populations of *B. tabaci* in the United States are almost exclusively MEAM1. Another principal *B. tabaci* is the Mediterranean (MED, formally called Q biotype) group, which likely originated from the Iberian Peninsula and has since spread globally [29,32,33]. To date, the genetic groups of *B. tabaci* consist of at least 40 morphologically indistinguishable cryptic species [20,30,31,34]. Moreover, the cryptic species of *B. tabaci* in some reports have not been identified. Thus, different cryptic species of *B. tabaci* in this review were all referred to as *B. tabaci*.

## 3. Biology

The life cycle of *B. tabaci* consists of egg, first nymphal instar, three additional nymphal instars (second, third, and fourth), and adult [35,36].

**Figure 1:**
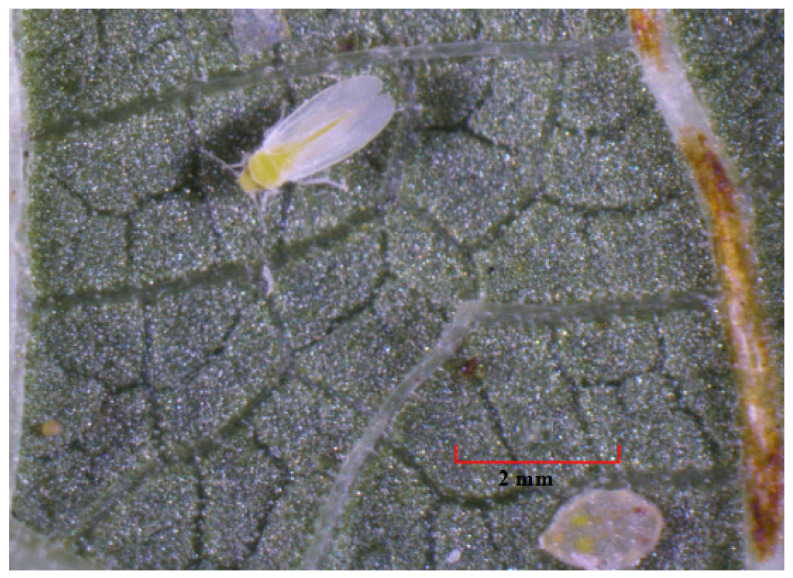
*Bemisia tabaci* egg (on low left side), adult and fourth instar nymph on the underside of a snap bean (*Phaseolus vulgaris* L.) leaf under microscope with 12.6× magnification (Li et al., unpublished).

**Figure 2:**
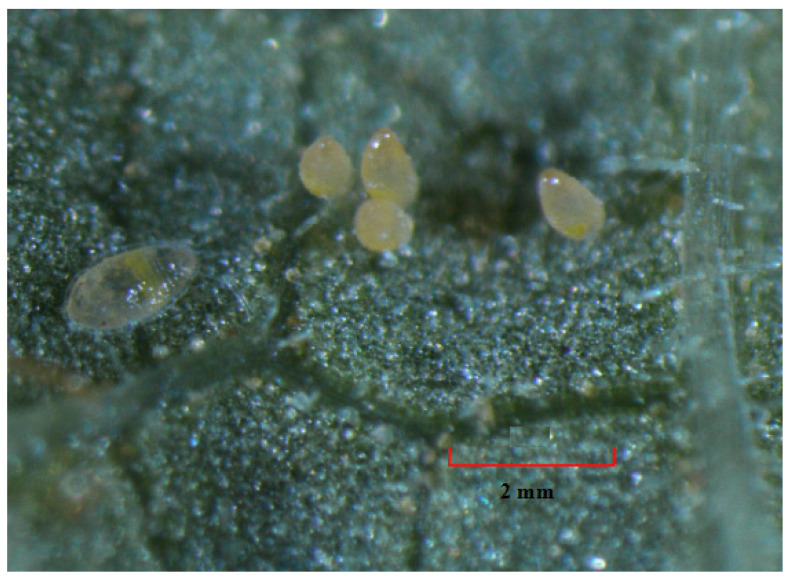
*Bemisia tabaci* eggs and first instar nymph on the underside of a snap bean leaf under microscope with 40× magnification (Li et al., unpublished).

Eggs: Female *B. tabaci* deposits approximately 0.2 mm long pear-shaped eggs into the mesophyll or inner tissue of the leaf [37]. Eggs are generally laid on the underside surface of the tender and upper leaves of the plants and are attached to the leaf by a stalk-like structure called the pedicel [38]. Eggs are pale yellow when first laid and become brown before hatching (Figure 1 and Figure 2).

Nymphs: *Bemisia tabaci* nymphs are oval, about 0.7 mm long for the fourth instar, and dorsoventrally flattened (Figure 1 and Figure 2) [35,37]. The early first instar or “crawler” is highly mobile after hatching and moves a short distance before settling to feed [36,39]. The lateral margins of the crawlers have many setae that are absent in later instars [35]. Once settled, they undergo ecdysis three times, and the resultant three nymphal stages are scale-like, sedentary, and possess greatly reduced legs and antennae [35]. The later developmental stage of the fourth nymphal instar is called “pupal stage” [40]. Before transition into an adult, externally observable characteristic of the fourth instar is the enlargement of the eyes from small red pinpoints to larger diffuse red oval spots, and finally to conspicuous red eye spots (Figure 1) [40]. Thus, the fourth instar in this period is also named “red-eyed nymph” (Figure 1) [40].

Adults: *Bemisia tabaci* females (about 0.94 mm long) are larger than the males (about 0.78 mm long) in size [41]. A *B. tabaci* adult possesses a pale-yellow body and two pairs of white wings covered with white powder wax [36,37]. At rest, the wings are held “roof-like” over the abdomen (Figure 1) [42]. Adults are typically found on the lower leaf surface of most plants [42].

A *Bemisia tabaci* female has a rounded abdomen, while the male abdomen is more pointed [42]. Moreover, *B. tabaci* adults possess arrhenotokous reproductive system, with unfertilized eggs developing into haploid males and fertilized eggs developing into diploid females [42]. The average longevity of adult females ranged from 44 days at 20 °C to 10 days at 35 °C [43,44,45,46].

The developmental time of *B. tabaci*, from egg to adult, is 14 to 105 days, but varies depending on temperature and host plant [43,44,47,48,49]. For instance, at 20–32 °C, development time ranged from 36.0 to 14.6 days on cantaloupe (*Cucumis melo* L. var. cantalupensis) and 37.9 to16.3 days on cotton (*Gossypium hirsutum* L.) [47]. Several studies on different crops have reported an optimal temperature range for *B. tabaci* growth as 20–33 °C [43,48].

The fecundity of *B. tabaci* varied from 324 eggs per female at 20 °C to 12.5 eggs per female at 37 °C [43,44,45,46]. *Bemisia tabaci* females were reported to be capable of laying over 500 eggs [50]. Host plants may influence the fecundity [46,51]. For example, at 30 °C, eggs per female ranged from 153.3 to 158.3 on cantaloupe, 117.0 to 117.5 on cotton, and 2.1 to 40.5 on pepper (*Capsicum annuum* L.) [47].

The survivorship of *B. tabaci* is also dependent on temperature and host plant [43,44,45,47,49]. The survivorship from egg to adult was 40%, 89%, and 37% at 15 °C, 25 °C, and 35 °C, respectively [43]. The survival rate of immature at 20 to 32 °C ranged from 100% to 76.5% on cantaloupe, 64.4% to 37.3% on cotton, and 8.3% to 0.0% on pepper [47].

## 4. Hosts and Plant Damage

*Bemisia tabaci* is among the most important insect pests worldwide in subtropical and tropical agriculture as well as in greenhouse production systems [37,52,53,54,55]. *Bemisia tabaci* is a highly polyphagous species which attacks over 1000 plant species belonging to 74 families [54,56,57,58]. The vegetable crops most affected by *B. tabaci* include bean (*Phaseolus vulgaris* L.), broccoli (*Brassica oleracea* L. var. italica), cabbage (*Brassica oleracea* L. var. capitata), cauliflower (*Brassica oleracea* L. var. botrytis), cucumber (*Cucumis sativus* L.), eggplant (*Solanum melongena* L.), melon (*Citrullus lanatus* L.), pepper, squash (*Cucurbita pepo* L.), tomato (*Solanum lycopersicum* L.), and watermelon (*Citrullus lanatus* L.) [37]. *Bemisia tabaci* also feed on agricultural crops, such as alfalfa (*Medicago sativa* L.), cotton, peanut (*Arachis hypogaea* L.), soybean [*Glycine max* L. Merr.], as well as ornamental plants, including poinsettia (*Euphorbia pulcherrima* Willd. ex Klotzsch), hibiscus (*Hibiscus rosa-sinensis* L.), and chrysanthemum (*Chrysanthemum morifolium* Ramat. Hemsl.). These agricultural crops and ornamental plants may serve as sources of *B. tabaci* for vegetable plantings [37,58].

*Bemisia tabaci* are sap-sucking insects with piercing–sucking mouthparts [2,35,41]. Infestation by *B. tabaci* may result in plant damage in four ways [35,41,59].

Firstly, *B. tabaci* nymphs and adults remove sap from phloem in plant stems and leaves [59,60]. Feeding damage caused by large populations may turn leaves yellow and dry, or even cause them to fall off plants [14]. Furthermore, heavy infestations of nymphs and adults could cause seedling death, stunting, or reduction in vigor and yield of older plants [14,61].

Secondly, *B. tabaci* nymphs and adults excrete honeydew, which renders leaves sticky and forms a substrate for the growth of black sooty mold [59,62]. The honeydew attracts ants, which impedes the activities of natural enemies of *B. tabaci* and other insect pests [63]. Moreover, the black sooty mold may create indirect damage by inhibiting plant respiration and photosynthesis [58].

Thirdly, *B*. *tabaci* has been labeled as a “supervector” because it transmits over 100 plant viruses, including Tomato yellow leaf curl virus (TYLCV), Tomato chlorosis virus (ToCV), Cucurbit yellow stunting disorder virus (CYSDV), Cucumber vein yellowing virus (CVYV), Squash vein yellowing virus (SqVYV), and Bean golden mosaic virus (BGMV) [37,64,65,66,67]. TYLCV is among the most devastating viruses that infect tomato crops worldwide [7,22,59,68,69].

Fourthly, *B*. *tabaci* adults and nymphs can inject salivary fluid during feeding, which may cause plant disorders [70]. Different physiological disorders induced by *B. tabaci* include silvering of squash [71,72,73], chlorotic streak of bell pepper (*Capsicum annuum* L.) [74], and irregular ripening of tomato [20,71,75].

These four types of plant damage can be severe when occurring alone or together. However, the hierarchy of *B. tabaci* damage potential is plant viruses > plant disorders > sap removal, leading to the reverse relationship for economic injury levels [76].

## 5. Incidence and Impact of *B. tabaci* on Vegetables in the Southern United States

*Bemisia tabaci* is a major insect pest in greenhouse-grown and field-grown vegetables in the United States [7,8,9]. This insect is a key pest of various vegetable crops in the southern states of the United States [62]. It has been found throughout the Southern United States and can survive in winter outdoors as far north as South Carolina [77]. Here, this concludes the incidence and impact of *B. tabaci* on vegetable crops in the Southern United States, including Alabama, Arkansas, Delaware, Florida, Georgia, Kentucky, Louisiana, Maryland, Mississippi, North Carolina, Oklahoma, South Carolina, Tennessee, Texas, Virginia, and West Virginia.

The first *B. tabaci* collected in the New World was discovered in 1894 in the United States on sweet potato and described as *Aleyrodes inconspicua* Quaintance, then named as sweetpotato whitefly [78]. Thereafter, *B. tabaci* had been a long-time resident in different agricultural regions throughout the southern and western states. However, it was not recognized as an economic pest until 1981 when fall vegetables, melon crops, and sugar beets were decimated in the Southwest United States by Lettuce infectious yellows virus (LIYV) transmitted by *B. tabaci* that was later designated “biotype A” (now called New World population) [15]. Soon after the introduction of *B. tabaci* MEAM1 into the United States around 1985, unprecedented losses started occurring on poinsettia in Florida in 1986, followed by high infestations in field-grown tomato crops [10,11,12]. The appearance of silverleaf symptoms on squash was also reported in Florida in 1986 [71,79]. In 1987, outbreaks of *B. tabaci* MEAM1 and two new disorders (squash silverleaf and tomato irregular ripening) occurred widely in South Florida [11]. Then *B. tabaci* MEAM1 rapidly spread across the Southern United States to Texas, Arizona, and California, where extreme field outbreaks occurred on melons, cotton, and other vegetable crops during the early 1990s [80,81,82,83]. Moreover, the begomovirus Tomato mottle virus (ToMoV) and TYLCV appeared in Florida in 1989 and 1994, respectively [76]. Notably, populations of *B. tabaci* were particularly problematic in the Southeastern United States in 2007 [54].

*Bemisia tabaci* is a significant threat to tomato production in fields in the Southeastern United States [7,22,68,69]. It can transmit phloem-limited TYLCV to tomato plants in a persistent–circulative manner [84,85,86,87]. TYLCV is now endemic throughout most of the Southern United States, including Florida, Georgia, North Carolina, Louisiana, and Texas [88,89,90].

### 5.1. Florida

Tomato is the most important vegetable crop in Florida, which leads all other states in fresh market production [91]. *Bemisia tabaci* has been a major pest of tomato plants since 1986. In Florida, the main whitefly-transmitted viral concerns are TYLCV, BGMV, SqVYV, Cucurbit leaf crumple virus (CuLCrV), and CYSDV [92]. Economic losses in the tomato industry from *B*. *tabaci* and associated geminivirus in Florida in 1991 were reported to exceed USD 125 million (Table 1) [93]. Direct damage, effects of the whitefly-borne tomato mottle geminivirus, and control costs in Florida were estimated at USD 141 million for the 1990–1991 seasons (Table 1) [14].

### 5.2. Georgia

*Bemisia tabaci* MEAM1 was confirmed from 1991 field collections from South Georgia where high populations were observed in peanut [101]. *Bemisia tabaci* and whitefly-transmitted viral diseases (e.g., CuLCrV, CYSDV, and TYLCV) pose a great threat to the economical production of vegetable crops, especially tomato, snap bean, and most cucurbit crops in Southern Georgia [102,103]. *Bemisia tabaci* outbreaks occurred on vegetables in Georgia in 2016 and 2017 [94,95]. Whitefly-transmitted viral diseases, such as CuLCrV and CYSDV, were particularly severe [94,95]. Economic losses associated with *B. tabaci* infestation and its viral transmitted diseases were substantial and estimated at approximately USD 132.3 and 161.2 million in 2016 and 2017, respectively (Table 1) [94,95]. Particularly, these viruses during the fall of 2017 caused 45% and 35% of the crop losses in snap bean and squash, respectively (Table 1) [94,95].

### 5.3. Texas

*Bemisia tabaci* is the most economically consequential whitefly species in Texas [104]. In a 1991 outbreak of *B. tabaci* in South Texas, the direct losses of vegetable were estimated at USD 29 million (Table 1) [96].

### 5.4. Arkansas

*Bemisia tabaci* has become increasingly crucial in cucurbit production in Arkansas. In years with hot and dry summers, there were numerous reports of *B*. *tabaci* infestations by cucurbit growers, especially on fall pumpkins (*Cucurbita maxima* Duchesne) [97]. *Bemisia tabaci* generally do not survive winters in Arkansas. *Bemisia tabaci* problems in fruiting vegetables (e.g., eggplant, okra (*Abelmoschus esculentus* L.), and pepper) in 2006 in Arkansas may have been correlated to the concurrence of several mild winters and hot–dry summers (Table 1) [97].

### 5.5. Kentucky

*Bemisia tabaci* is a relatively new pest to Kentucky and has been observed to attack squash and tomato [98,105]. Though it has been a problem in the southern regions of the United States for several years, *B. tabaci* outbreaks in Kentucky had been relatively uncommon [98]. However, problems were observed during the dry hot summer of 2007 in tomato heirloom varieties (Table 1) [98]. *Bemisia tabaci* infests greenhouse-grown plants throughout the year in Kentucky but is unable to survive winters in the crop fields [98].

### 5.6. Tennessee

*Trialeurodes vaporariorum* is the most common species infesting tomato in Tennessee, while *B. tabaci* sometimes infests greenhouse-grown tomato. However, *B. tabaci* infestations in field-grown tomato have been sporadic in 2014 in Tennessee; less than 5% of fields have problems (Table 1) [99]. The most critical damage is sooty mold growth on honeydew, which accumulates on fruits and leaves, reducing photosynthesis [99].

### 5.7. South Carolina

*Bemisia tabaci* is a sporadic problem in vegetables in South Carolina. An outbreak of *B*. *tabaci* in 2017 was unusually severe and widespread (Table 1) [100].

### 5.8. Other States

Compared to the aforementioned states, the impact of *B*. *tabaci* on field-grown vegetables has been relatively less severe in other states in the Southern United States (e.g., Alabama, Delaware, Louisiana, Maryland, Mississippi, North Carolina, Oklahoma, Virginia, and West Virginia). Thus, minimal documentation is available.

## 6. Management

Extensive economic losses owing to the plant damage and control efforts of *B. tabaci* have resulted in the acceleration of research to provide potential management strategies [41,106]. This review discusses the common control measures (e.g., cultural, chemical, and biological control, as well as host-plant resistance) on vegetables used mainly in the Southern United States, but relevant information from other regions of the world is included.

### 6.1. Monitoring and Sampling

Efficient, accurate, and practical monitoring and sampling methods are crucial to the implementation of any management strategy [107,108] because those tools provide consistent and reliable means for measuring pest density and determining the need for control [50]. Numerous studies have concentrated on determining the monitoring and sampling methods for *B*. *tabaci* on vegetable crops, such as tomato [109,110,111,112,113], cucumber [114], watermelon [115,116,117], muskmelon (*Cucumis melo* L.) [116], melon [118,119], common bean (*Phaseolus vulgaris* L.) [120,121], eggplant [116], and paprika (*Capsicum annuum* L.) [122]. Most of the research has compared various methods and techniques for determining relative density (e.g., on-plant counts, beat trays, and sticky traps); describing the within and between plant distributions of different life stages; selection of optimal sample units, sizes, and sampling techniques; as well as establishment of various economic injury levels and sampling plans (sequential and binomial).

The yellow sticky trap is among the most widely used methods, especially in larger-scale monitoring of *B*. *tabaci* incidence. However, due to the lack of correlation between trap catches and field populations, the yellow sticky trap may not be utilized when estimating *B*. *tabaci* density in either research or pest management application [123,124,125].

Progress has been made in automating insect counting that could reduce effort and increase accuracy in monitoring and sampling insect pests [122,126,127,128,129]. For instance, automating the count of whitefly nymphs on leaves has been investigated with digital video and image processing [126]. A prototype system was developed to automate the counting of adult *T. vaporariorum* and *B. tabaci* using digital image analysis [127]. More recently, a novel counting algorithm has been explored for estimating whiteflies on vegetables (cayenne pepper (*Capsicum annuum* L.), cucumber, tomato, and eggplant) utilizing computer images with high accuracy [128].

### 6.2. Cultural Control

Cultural control relies on the manipulation of production practices to render the environment unfavorable to pests [41,130].

Water and fertility management plays a vital role as a cultural tactic in *B. tabaci* control on vegetable plants [131,132,133,134,135,136,137]. For instance, compared to furrow and sprinkler irrigation, daily drip irrigation resulted in the lowest *B. tabaci* density and incidence of whitefly-transmitted viruses in cucumber, green bean, squash, and tomato [132]. However, when integrating with the tomato–coriander (*Coriandrum sativum* L.) intercropping strategy, sprinkler irrigation reduced *B. tabaci* and associated viruses on organic tomato crops [135]. Moreover, increasing nitrogen levels increased *B. tabaci* populations in okra [131] and hydroponic tomato [136]. Several sulfur-containing fertilizers at different rates were studied in 10 vegetable crops and were demonstrated to have mixed effects on *B. tabaci* populations [137].

One important cultural control strategy is to increase host-free periods or reduce intercrop migrations [138,139,140]. Altering planting dates is one means to provide host-free periods between successive crops [141]. The incidence of *B*. *tabaci* and associated viruses on tomato and other vegetable crops in Florida was greatly reduced by maintaining fields free of host crops for at least two months during the rainy summer period [139]. However, no benefit was observed regarding *B. tabaci* abundance and associated virus incidence by planting cucumber, squash, and tomato one month earlier than standard planting dates in a study in Egypt [142].

Living mulches (a cover crop interplanted or undersown with a main crop and intended to serve the purposes of a mulch) and synthetic mulches have been adopted for mitigating *B. tabaci* problems on vegetables [143,144,145,146]. Living mulches are effective in reducing the density of *B. tabaci* and the incidence of associated viruses in tomato [145] and zucchini squash (*Cucurbita pepo* L.) [147,148]. Synthetic reflective mulch resulted in a lower incidence of *B. tabaci* and related viruses in watermelon [143], zucchini squash [147], snap bean [149], and tomato [144,150]. Silver plastic mulch effectively decreased the incidence of TYLCV in tomato fields in Florida [151]. Furthermore, it was indicated that zucchini squash infested by *B. tabaci* within synthetic UV reflective mulches obtained significantly higher yields than those grown with living mulch of buckwheat (*Fagopyrum esculentum* Moench) in Florida [146].

In protective structures (such as greenhouses and screenhouses), *B. tabaci* can be physically excluded from the vegetable crops when using fine mesh materials [152,153]. However, a conflict exists between adequate ventilation and the small mesh size required for the exclusion of *B. tabaci*. Thus, an electric field screen has proven successful in the “repel and/or capture” of *B. tabaci* on tomato crops in ventilated greenhouses [154,155,156,157,158,159].

UV-absorbing materials have been applied in high tunnels and other forms of protected culture to reduce *B. tabaci* populations on vegetables [160,161,162,163]. UV-absorbing plastic sheets/film cover could significantly decrease *B. tabaci* populations and the incidence of whitefly-borne viruses on tomato crops [160,161,162]. It was reported that the virus infection levels in tomato were only 6%–10% in UV-blocking greenhouses compared with 96%–100% in UV-nonblocking greenhouses [163].

Trap crops (preferred host plants which are used to draw a herbivore away from a less-preferred main crop) [164] and barrier crops are among the cultural control methods promoted for *B. tabaci* management. Studies associated with trap crops and barrier crops have mixed results. Squash, cucumber, and eggplant have been shown to be promising trap crops for protecting tomato [165,166,167,168] and snap bean [149] against *B. tabaci*. Maize (*Zea mays* L.) was demonstrated to be a good barrier crop of tomato [168]. Conversely, it was revealed that eggplant or corn as a trap crop did not reduce *B. tabaci* populations on tomato crops [169]. Moreover, neither *B. tabaci* egg nor nymphal densities on common bean leaves were reduced by the trap crop eggplant or the barrier crop corn in North Florida [170].

Intercropping to prevent *B. tabaci* from locating host plants has shown promise. Lower *B. tabaci* densities and/or incidence of associated viruses were observed on tomato intercropped with coriander [135,145,171], squash [172], maize [142], chili pepper (*Capsicum annuum* L.) [173], cucumber [169,174], French bean (*Phaseolus vulgaris* L.) [175], onion (*Allium cepa* L.) [176], and garlic (*Allium sativum* L.) [176]. Additionally, intercropping okra with coriander or ginger (*Zingiber officinale* L.) has been reported as one alternative strategy to suppress *B. tabaci* populations in okra [177,178]. Zucchini intercropped with okra had lower numbers of adult *B. tabaci* and a lower severity of squash silverleaf disorder compared with non-intercropped zucchini [148]. By intercropping cucumber with lettuce (*Lactuca sativa* L.), the number of *B. tabaci* adults on cucumber leaves was reduced by 69.7% [179].

### 6.3. Host-Plant Resistance

Host-plant resistance is an essential strategy in successful IPM program for the suppression of *B. tabaci* populations [180].

In tomato, numerous efforts for breeding resistance to *B. tabaci* have been extensively implemented. *Bemisia tabaci* exhibited reduced host preference (antixenosis) and reproduction (antibiosis) on tomato cultivars with *Mi* gene (a broad-spectrum resistance gene, which encodes a coiled-coil, nucleotide-binding, leucine-rich repeat receptor) [181,182,183]. For example, the tomato gene *Mi-1.2* has been shown to confer resistance to *B. tabaci* by decreasing infestation, oviposition, and the number of fourth instar nymphs [184]. The types of tomato trichomes play a critical role in resistant tomato varieties. It was observed that the tomato variety Martha with a high density of glandular trichomes was moderately resistant to *B. tabaci* [185]. In the wild tomato, *Solanum peruvianum* (reported as *Lycopersicon hirsutum*), the elevated type IV glandular trichomes (one of four types of glandular trichomes (types I, IV, VI, and VII)) were found to be highly correlated with a reduction in *B. tabaci* infestation [186]. Nevertheless, the results were not consistent, as some studies revealed that higher densities of non-glandular trichomes could be associated with increased oviposition rates by *B. tabaci* [187,188]. Moreover, the development of *B. tabaci* resistance in tomato has concentrated on screening existing tomato varieties [189] or their wild relatives of cultivated tomato [187,190,191,192,193]. Newer tomato varieties, such as Charger, Rally, and Tygress, have been discovered to support significantly season-long low densities of *B. tabaci* eggs and nymphs in Florida [189].

Resistance against *B. tabaci* and associated viruses was also detected in many additional cultivated vegetable crops, such as watermelon [143,194], mung bean (*Vigna radiata* L.) [195,196], chili pepper [197,198,199], pepper accessions (*Capsicum annuum* L., *C. chinense* Jacq., and *C. baccatum* L.) [200], and collard (*Brassica oleracea* L. var. viridis) [201,202]. A perennial desert species of *Citrullus ecirrhosus* was observed to exhibit resistance against *B. tabaci*, and thus became exploited as a source of *B. tabaci* resistance in breeding cultivars of watermelon [194]. Two mung bean varieties, SML-668 and Pant Moong-1, have been identified to be moderately susceptible to *B. tabaci* and Mungbean yellow mosaic virus (MYMV) [196]. Eighty-two commercial snap bean lines were screened for two years and two varieties were identified that consistently displayed moderate levels of resistance to both CuLCrV and Sida golden mosaic virus (SiGMV) transmitted by *B. tabaci* (Bhabesh Dutta; Personal communication) [203]. Chili pepper genotypes of IHR 4283, IHR 4329, IHR 4300, IHR 4321, and IHR 4338 were reported to harbor low numbers of *B. tabaci* adults and nymphs [199]. The accessions IAC-1544 (*C. annuum*), IAC-1545 (*C. chinense*), and IAC-1579 (*C. annuum*) could be classified as good sources of resistance against *B. tabaci* and associated virus Tomato severe rugose virus (ToSRV) for cultivated pepper [200]. Collard greens genotypes VE and J exhibited high resistance against *B. tabaci* [202].

Transgenic vegetable plants that provide resistance to *B. tabaci* and/or associated viruses have been developed, such as transgenic tomato [204,205,206,207,208,209,210], transgenic bean [211,212,213], and transgenic lettuce [214]. For example, transgenic tomato plants were screened for resistance to TYLCV, and it was demonstrated that no TYLCV symptoms were observed and no TYLCV genomic DNA was detected in the progeny of TYLCV transgenic tomato plants [208]. After *B. tabaci* fed on genetically modified bean for 8 days, the BGMV DNA amount in *B. tabaci* was significantly reduced 84% [213]. Moreover, it was observed that within 5 days of feeding on genetically engineered lettuce, *B. tabaci* showed a mortality rate of up to 98.1% [214].

### 6.4. Chemical Control

Applications of chemical insecticides are among the main tools used commonly by vegetable growers to control *B. tabaci*. Heavy reliance on insecticides against *B. tabaci* is still widespread in open field vegetable crop systems [41,59]. The systemic neonicotinoids play a crucial role, particularly those that are relatively stable in the soil and effectively absorbed through the root system. A group of insecticides with other modes of action has been widely utilized against *B. tabaci*, such as the insect growth regulators (pyriproxyfen and buprofezen), ketoenols (spiromesifen and spirotetramat), and diamides (anthranilic diamides, cyantraniliprole, and chlorantraniliprole) [41,59]. Oils, soaps, and detergents have also been applied widely for *B. tabaci* control [41,59].

#### 6.4.1. Neonicotinoid Insecticides

The neonicotinoids are among the most effective group of insecticides. Imidacloprid was the first commercial neonicotinoid, eventually becoming the most widely used insecticide worldwide [215]. Imidacloprid is effective against sucking insect pests, including *B. tabaci* [216]. The application of imidacloprid on vegetables and melons was a key chemical tactic in the United States [138]. Imidacloprid has widespread use for the management of *B. tabaci* on vegetables in Florida [217,218], and its application resulted in dramatic declines in *B. tabaci* populations on tomato plants in Southwest Florida during the period 1994–1996 [217]. Imidacloprid has been extensively applied against *B. tabaci* on vegetables in Texas as well [219,220]. It was demonstrated that imidacloprid provided exceptionally good control of *B. tabaci* on cabbage and cantaloupe in Texas [219].

#### 6.4.2. Insect Growth Regulators

Pyriproxyfen, a juvenile hormone analog, has been utilized to successfully control *B. tabaci* in cotton [221,222,223]. Buprofezin, a chitin inhibitor, has been widely used against *B. tabaci* on cotton crop in the Southwestern United States [223]. Buprofezin has been labeled against *B. tabaci* on melons, and pyriproxyfen has the potential to be labeled against *B. tabaci* on melons and vegetables [220]. It was revealed that three biweekly applications of insect growth regulators (pyriproxyfen and buprofezin) and five biweekly applications of fungal insecticides (Mycotrol^®^ and Naturalis-L^®^), plus one soil application of imidacloprid, significantly reduced *B. tabaci* populations on spring cantaloupe in Texas [220].

#### 6.4.3. Ketoenols

Ketoenols are a relatively new class of insecticides that are derivatives of tetronic acids (spiromesifen) and tetramic acids (spirotetramat) [224]. These compounds act as inhibitors of lipid biosynthesis by targeting acetyl-CoA carboxylase and adversely influence juvenile stages with additional effects on adult fecundity [225,226]. Spiromesifen and spirotetramat have been widely applied to effectively control *B. tabaci* on vegetable crops [227,228,229,230,231]. Spiromesifen was found to be highly toxic to *B. tabaci* on melon and collard with almost 100% nymphal mortality in laboratory bioassays and exhibit excellent promise against *B. tabaci* populations on melon and collard in field trials [228]. It has also been reported that spirotetramat is superior against *B. tabaci* on bell pepper compared to the neonicotinoid insecticide of imidacloprid 200 SL [230].

#### 6.4.4. Diamides

Diamide insecticides are the most recent chemistry class in the market [232] and act on insect ryanodine receptors causing mortality by the uncontrolled release of calcium ion stores in muscle cells [233]. Cyantraniliprole, a commercialized diamide insecticide, targets sucking pests, such as *B. tabaci* [234]. Cyantraniliprole has been demonstrated to be effective against *B. tabaci* in tomato [235,236,237,238,239]. For instance, cyantraniliprole applied as soil treatments or foliar sprays provided excellent adult *B. tabaci* control, TYLCV suppression, as well as reduced oviposition and nymph survival on tomato [238].

#### 6.4.5. Soaps and Oils

Soaps and oils have been long used as insecticides [240]. Both soaps and oils function through blocking the spiracles, oils with a thin oil film, and soaps with super-wetted water [241]. Oils are also known to reduce the settling of *B. tabaci* on plants and influence the transmission of plant viruses [242]. Soaps and oils have been proven to provide excellent efficacy in suppressing *B. tabaci* populations on vegetables, such as tomato [243,244,245,246,247], cucumber [243], zucchini squash [243], collard [248], and melon [249]. For example, an insecticidal soap, two horticultural oils, and 12 detergents resulted in >85% mortality of *B. tabaci* nymphs in zucchini squash and tomato under greenhouse conditions; and Saf-T-Side (mineral) Oil^TM^, Natur’l^TM^ (vegetable) oil, New Day^TM^ liquid detergent or M-Pede^TM^ reduced the number of *B. tabaci* adults on heavily infested cucumber plants in field in Florida [243].

#### 6.4.6. Plant-Based Products

Various plant-based products have been extensively investigated for their activity against *B. tabaci* on vegetable crops [250,251,252,253]. Plant extracts were indicated to adversely impact *B. tabaci* populations, including neem-based products [254,255,256,257,258], extracts from milkweed (*Calotropis procera* (Aiton) W.T. Aiton) and garlic [259], extracts from *Jatropha curcas* L. [260], as well as fermented extracts of neem and wild garlic (*Tulbaghia violacea* Harvey) [261]. A drench application of NeemAzal, a commercial neem-based product, resulted in up to 90% mortality of immature *B. tabaci* populations in tropical open field-grown and greenhouse-grown tomato [262]. Moreover, plant-derived essential oils have also been widely evaluated to manage *B. tabaci* [263,264,265,266,267,268,269]. For example, essential oils of *Piper* callosum, *Adenocalymma alliaceum*, *Pelargonium graveolens*, and *Plectranthus* neochilus deterred the settlement and oviposition of *B. tabaci* adults on tomato plants [266].

### 6.5. Insecticide Resistance and Its Management

A review of insecticide resistance in *B. tabaci* summarized various resistance mechanisms, several bioassays used to evaluate resistance, studies for monitoring resistance, known cases of insecticide resistance, and some strategies that can be used to mitigate resistance [270]. More recently, another review also concluded insecticide resistance and its management issues among *B. tabaci* species but focusing mainly on research published from 2010 to 2020 [59]. Their review proposed several tactics for alleviating the resistance in *B. tabaci*, namely, chemical control with selective insecticides, rotation of insecticides with different modes of action, insecticide mixtures, and nonchemical control methods (e.g., cultural control, host-plant resistance, and biological control methods) [59].

### 6.6. Biological Control

Biological control plays an essential role in *B. tabaci* IPM systems. There are at least 48 species of predators, 62 species of parasitoids, and 9 species of pathogens reported as natural enemies of *B. tabaci* [37,271]. The natural enemies associated with *B. tabaci* include the predators in the following genera: *Brumoides*, *Verania*, *Cheilomenes*, *Macrolophus*, *Nesidiocoris*, *Geocoris*, *Orius*, *Chrysopa*, *Coccinella*, and *Amblyseius*; the parasitoids in the genera *Encarsia* and *Eretmocerus*; the nematodes in the genera *Steinernema* and *Heterorhabditis*; as well as the fungi in the genera *Beauveria*, *Cordyceps* (formerly known as *Isaria* or *Paecilomyces*), and *Verticillium* [272].

Numerous studies have been conducted and reviewed on the distribution, life history, bionomics, and ecology of natural enemies for *B. tabaci* management [271,273,274,275,276]. The focus here is the efficacy of natural enemies against *B. tabaci* on vegetable crops over the past two decades.

#### 6.6.1. Predators

Although over 150 arthropod species have been described as predators of *B. tabaci*, only a few have been studied thoroughly and many are still under limited laboratory observations or qualitative field records [271,273]. Most of the known predators of *B. tabaci* are coccinellid beetles, predaceous bugs, lacewings, and phytoseiid mites.

The predatory *Harmonia axyridis* Pallas (Coleoptera: Coccinellidae) was demonstrated to suppress *B. tabaci* in greenhouse-grown tomato when released with a parasitoid, *Encarsia formosa* Gahan or *Encarsia sophia* Girault and Dodd (Hymenoptera: Aphelinidae) [277]. *Delphastus catalinae* Horn (Coleoptera: Coccinellidae) is the most commonly used predacious natural enemy and commercially available for *B. tabaci* control on various vegetable crops in greenhouses [41]. For example, significant reductions in *B. tabaci* populations on tomato plants were attributed to the release of *D. catalinae* [278]. This beetle originates from tropical regions, but wild populations exist in Central and Southern Florida [279]. Field populations of *D. catalinae* are not known to occur in Northern Florida, Georgia, or South Carolina [279].

In vegetable greenhouses, successful biological control of *B. tabaci* using mirids includes the inoculative and augmentative release of *Macrolophus pygmaeus* Rambur (Hemiptera: Miridae) and *Nesidiocoris tenuis* Reuter (Heteroptera: Miridae). For instance, *M. pygmaeus* could control *B. tabaci* populations in melon when released at 6 adults/plant into the initial infestation of 10 *B. tabaci* adults/plant in greenhouses [280]. Two release rates of *N. tenuis* (1 and 4 individuals/plant) resulted in a >90% reduction in *B. tabaci* populations in tomato under greenhouse conditions [281]. However, *N. tenuis* did not contribute to *B. tabaci* suppression in protected sweet pepper (*Capsicum annuum* L. (Solanaceae) cv. Spiro) [282]. Predation on *B. tabaci* was compromised when *M. pygmaeus* and *N. tenuis* coexisted on tomato plants but was improved when the parasitoid *Eretmocerus mundus* Mercet (Hymenoptera: Aphelinidae) was added into the mix [283]. Additionally, combining another mirid predator, *Macrolophus melanotoma* Costa (Hemiptera: Miridae), and the parasitoid *Er. mundus* supported a low number of *B. tabaci* in greenhouse-grown eggplant [284].

The release of the lacewing *Chrysoperla rufilabris* Burmeister (Neuroptera: Chrysopidae) provided significant *B. tabaci* suppression on watermelon in the field, but no effect in a legume field [285]. The predatory efficiency of another lacewing *Chrysoperla carnea* Stephens (Neuroptera: Chrysopidae) on *B. tabaci* in greenhouse-grown tomato was also investigated, and the lowest number of *B. tabaci* was observed at a release rate of 10 *C. carnea* larvae/plant [286]. Furthermore, the reduction in *B. tabaci* in squash was >90% after a third predator release of 10 *C. carnea* larvae/plant and reached 100% after six releases [287]. *Bemisia tabaci* populations on sweet pepper plants were reduced about 97% after the second predator release of 10 *C. carnea* larvae/plant [287]. Integrating *C. carnea* with the predatory bug *Orius albidipennis* Reuter (Hemiptera: Anthocoridae) and the predatory mite *Phytoseiulus persimilis* Athias-Henriot (Acarina: Phytoseiidae) decreased *B. tabaci* populations and increased cucumber yields in greenhouses [288].

The predatory mite *Amblyseius swirskii* Athias-Henriot (Acarina: Phytoseiidae) is considered among the most effective natural enemies and utilized extensively on vegetable crops [289]. Under semi-field conditions, *A. swirskii* at two release rates of 25 and 100 mites/m^2^ suppressed *B. tabaci* almost totally when an initial infestation was 8 *B. tabaci* adults per sweet pepper plant [290]. Similarly, *A. swirskii* provided a significant reduction in the *B. tabaci* populations in sweet pepper in greenhouses [282,291]. Moreover, the combination of 50 *A. swirskii*/m^2^ and 12 *Er. mundus*/m^2^ was the most efficient strategy in protected sweet pepper crops at an initial infestation of 50 adults of *B. tabaci* per plant [290]. Integrating the predatory mirids *N*. *tenuis* or *M*. *pygmaeus* with *A. swirskii* significantly suppressed *B. tabaci* populations on sweet pepper under greenhouse conditions [292].

The predatory mite *Amblyseius barkeri* Hughes or *Amblyseius cucumeris* Oudemans (Acarina: Phytoseiidae) suppressed *B. tabaci* populations on tomato plants in greenhouses [293]. Another predatory mite, *Amblyseius tamatavensis* Blommers (Acarina: Phytoseiidae), was first reported in 2018 in Southern Florida [294] and has been found to result in a 60% to 80% reduction in *B. tabaci* populations on bell pepper plants under laboratory conditions [295].

#### 6.6.2. Parasitoids

Parasitoids as biocontrol agents play a crucial role in IPM program of *B. tabaci*. The majority parasitoids of *B. tabaci* are from the genera *Encarsia* and *Eretmocerus* (Hymenoptera: Aphelinidae) [41,59,271], which are also the most common parasitoids attacking *B. tabaci* in Florida [92]. *Encarsia formosa*, *Eretmocerus eremicus* Rose and Zolnerowich (Hymenoptera: Aphelinidae), and *Er. mundus* have been extensively investigated for *B. tabaci* control [41,293].

*Encarsia formosa* is the most widely used parasitoid for the biological control of *B. tabaci* on greenhouse-grown vegetable crops [41]. For instance, releases of low numbers of *En. formosa* and *Er. mundus* resulted in *B. tabaci* parasitism of 62.0% and 77.9%, respectively, in sweet potato (*Ipomoea batatas* L.) field [296].

*Eretmocerus* species (especially *Er. eremicus* and *Er. mundus*) have recently gained important focus in the biological control of *B. tabaci* [297,298,299,300]. For example, *Er. eremicus* significantly suppressed *B. tabaci* eggs and nymphs in mint under greenhouse conditions [301,302]. Moreover, *Er. mundus* provided high incidence of parasitism and significant control of *B. tabaci* in sweet pepper and tomato in greenhouses [299,300,303,304]. Integrating *Er. mundus* with *A. swirskii* or *M. caliginosus* greatly reduced *B. tabaci* populations on greenhouse-grown sweet pepper and tomato plants [282,303].

#### 6.6.3. Entomopathogens

Entomopathogens (e.g., fungi, viruses, nematodes, protists, and bacteria) as biological control agents are a major component of IPM programs in regulating populations of insect pests [305,306,307]. Entomopathogens are viable alternatives to chemical insecticides due to minimal adverse effects on humans, other non-target organisms, and the environment [308,309], as well as delaying the development of insecticide resistance in pest populations by reducing insecticide inputs [310].

Entomopathogenic nematodes in the genera *Steinernema* and *Heterorhabditis* are important biological control agents that are employed to control various destructive insect pests [306,311,312]. These nematodes are obligate parasites in nature and kill insects with the aid of mutualistic bacteria [313]. Entomopathogenic nematodes play an essential role in *B. tabaci* management on vegetable crops [314,315,316,317,318]. For example, the applications of *Steinernema feltiae* Filipjev (Rhabditida: Steinernematidae) alone or combined with the insecticide imidacloprid significantly reduced *B. tabaci* survival in tomato and verbena (*Verbena officinalis* L.) [314,315,316,317]. Similarly, integrating the entomopathogenic nematode *Steinernema carpocapsae* Wesier (Rhabditida: Steinernematidae) with the insecticides thiacloprid or spiromesifen provided high *B. tabaci* mortality of 86.5% and 94.3%, respectively, on tomato plants [318]. Furthermore, the entomopathogenic nematodes *Heterorhabditis bacteriophora* Poinar (Rhabditida: Heterorhabditidae) Guatemala strain; *H. bacteriophora* Chiapas strain; as well as *Heterorhabditis indica* Poinar, Karunakar, and David (Rhabditida: Heterorhabditidae) native strain from Sinaloa, Mexico killed up to 95% of *B. tabaci* nymphs on tomato leaves under laboratory conditions [319]. However, the efficacy was not consistently high across studies. For example, mortality of first and second nymphal stages of *B. tabaci* in pepper caused by entomopathogenic nematodes was reported to be very low: 17.44% for *H. bacteriophora*, 35.26% for *S. carpocapsae*, and 18.48% for *S. feltiae* [320]. The application of *S. feltiae* only resulted in 32% and 28% *B. tabaci* mortality on tomato and cucumber plants, respectively [321].

Entomopathogenic fungi are also major components of plant protection programs for managing economically important insect pests [276,310,322,323,324,325]. Most entomopathogenic fungi infect their insect hosts by directly penetrating the cuticle [276]. Over 20 species of entomopathogenic fungi are known to infect whiteflies [106,326,327]. *Beauveria bassiana* (Balsamo-Crivelli) Vuillemin; *Cordyceps fumosorosea* (Wize) Kepler, B. Shrestha and Spatafora (formerly known as *Isaria fumosorosea* (Wize) or *Paecilomyces fumosoroseus* (Wize) A.H.S. Brown and G. Smith); and *Lecanicillium lecanii* (Zare and Gams) are the most widely investigated entomopathogenic fungi that infect *B. tabaci* on vegetable crops [41].

At 8 days post-inoculation of *B. bassiana*, the mean mortality of the fourth instar *B. tabaci* nymphs on cucumber was 91.8% [328]. Similarly, at 10 days post-inoculation of *B. bassiana*, the survival rate of nymphal *B. tabaci* was 4.2%, 9.6%, 13.4%, and 24.3% on cucumber, eggplant, tomato, and cabbage, respectively [329]. Furthermore, multiple applications of *C. fumosorosea* (reported as *P. fumosoroseus*) and *B. bassiana* provided >90% control of *B. tabaci* nymphs on cucumber, cantaloupe melon, and zucchini squash in small-scale field trials [330]. The isolates of *B. bassiana* and *C. fumosorosea* (reported as *I. fumosorosea*) also resulted in 71%–86% mortality of *B. tabaci* nymphs on bean leaves under laboratory conditions [331]. A follow-up screenhouse study revealed that the efficacy of *B. bassiana* and *C. fumosorosea* (reported as *I. fumosorosea*) against *B. tabaci* on common bean plants was improved significantly when mixed with the non-ionic surfactant Silwet^®^ L-77 [332].

The effect of integrating entomopathogenic fungi with chemical insecticides in suppressing *B. tabaci* populations has also been evaluated. The individual application of the entomopathogenic fungus *Lecanicillium muscarium* (Petch) Zare and Gams or integrating with the insecticide imidacloprid resulted in high mortality of *B. tabaci* on tomato, verbena, and cucumber plants under both laboratory and glasshouse conditions [333,334,335]. When *C. fumosorosea* (reported as *P. fumosoroseus*) and the insecticide azadirachtin were combined, up to 90% *B. tabaci* nymphal mortality was obtained, although the combined effects were less than additive effects [336]. Another entomopathogenic fungus, *Cordyceps javanica* (Frieder. and Bally) Kepler, B. Shrestha and Spatafora (reported as *Isaria javanica* (Friedrichs and Bally) Samson and Hywel-Jones) isolate caused up to 62.4% nymphal mortality of *B. tabaci* on bean plants, and an additive interaction was found when *C. javanica* (reported as *I. javanica*) was combined with the insecticides buprofezin or spiromesifen [337]. However, antagonism was observed when *B. bassiana* was integrated with imidacloprid against *B. tabaci* in cantaloupe melon [338].

## 7. Future Challenges and Opportunities

Although the aforementioned control measures can impact *B. tabaci* populations if applied independently, they could be more effective at reducing losses due to *B. tabaci* infestations when used in combination as part of an IPM program [220,318,336,337]. Different strategies will be imperative for different systems, growing conditions, and geographical areas. Greenhouse growers may take advantage of the enclosed environment by using screens to exclude *B. tabaci* [152,153] and by using predators [278,280,287,292], parasitoids [296,299,300,301,302,303,304], and entomopathogens [316,332]. Under field conditions, one general approach is to apply: 1) cultural practices to avoid or reduce *B. tabaci* infestations [139,148]; 2) biologically mild treatments (insecticidal soaps/oils or highly selective insecticides) to suppress *B. tabaci* populations while preserving beneficial organisms [243]; and 3) broad-spectrum pesticides only when required (based on action thresholds) preferably at the later stages of vegetable crops to minimize detrimental effects on beneficial organisms [217,220].

Vegetable breeding for resistance to both *B. tabaci* and associated viruses plays an important role in *B. tabaci* management [180]. New and more tolerant or resistant vegetable varieties have been successfully developed and will become commercially available in the future. Although substantial progress has been made in vegetable breeding for resistance against *B. tabaci* and associated viruses utilizing genetic engineering technology, the cultivation and acceptance of genetically engineered vegetable crops tend to be deferred for the future considering ethical and legislative issues.

The application of chemical insecticides remains the principal approach to suppress *B. tabaci* populations on vegetables [41,59]. Nevertheless, owing to the intensive selection pressure associated with the insecticide applications, *B. tabaci* populations have developed considerable resistance to insecticides from different chemical classes [59]. The chemical control approach has also caused resurgence of *B. tabaci*, secondary pest outbreaks, and environmental degradation [339]. Furthermore, the introduction of insecticides containing new active ingredients is limited due to costs affiliated with development and registration [340]. Additionally, increasing global warming may exacerbate *B. tabaci* problems, as it was noted that *B. tabaci* survived long-term under high temperature stress [341].

Successful biological control has been achieved in suppressing *B. tabaci* populations. There is a general trend in using two or more natural enemies together [277,283,284] or combining natural enemies with chemical insecticides [333,336,337]. The technologies of identification, mass production, and quality control of predators and parasitoids need to be elevated in the future. Moreover, further advancements in technologies are crucial for mass production, stabilization, formulation to avoid damaging environmental conditions (UV radiation and desiccation), and delivery of entomopathogens. More compatibility testing of natural enemies with each other and/or with chemical insecticides will be imperative to facilitate the incorporation of natural enemies into *B. tabaci* IPM systems.

Novel technologies provide more opportunities for *B. tabaci* management. Hyperspectral imaging along with machine-learning-based assessments might allow the rapid and accurate detection of *B. tabaci* on vegetables [122,126,127,128,129]. Although these systems are currently at a proof-of-concept stage, they promise possibilities for the efficient detection of *B. tabaci* infestations, especially at low densities. Subsequently, precision application of therapeutic management tactics, including insecticides, may be automated and coupled to monitoring systems, which makes them more practical for large-scale farming systems [342]. The precision management could decrease insecticide inputs, reduce product costs, expose workers less to insecticides, preserve natural enemies, and increase sustainability of pest management programs.

Among the potential novel technologies that can be used as tools for *B. tabaci* management, the biotechnological tool RNA interference (RNAi) is initiated by the introduction of double-stranded RNA into a cell, which results in the generation of loss-of-function phenotypes via the depletion of the target gene messenger [343,344]. RNAi technology can specifically silence the function of vital genes in insect pests [345,346,347]. RNAi has been well established in *B. tabaci* through injection [348,349], oral route [350], or by expressing its homologous double stranded RNA in plants [351]. To date, several genes have been targeted in *B. tabaci* through RNAi, and these studies demonstrated its potential to manage *B. tabaci* at the laboratory level [352,353,354]. Therefore, further research and investments may be required to move toward the field application.

## Figures and Tables

**Table 1:** Incidence and impact of *B. tabaci* on vegetable crops in the Southern United States.

Southern States	Time Frames	Vegetable Crops	Losses		Sources
			USD (Millions)	% of Crop	
Florida	1991	Tomato	>125		[93]
	1990–1991	Tomato	141		[14]
Georgia	2016	Tomato, snap bean, most cucurbit	132.3		[94,95]
	2017	Tomato, snap bean, most cucurbit	161.2		[94,95]
	2017	Snap bean		45	[94,95]
	2017	Squash		35	[94,95]
Texas	1991	Vegetables	29		[96]
Arkansas	2006	Eggplant, okra, pepper	Problems were observed		[97]
Kentucky	2007	Tomato	Problems were observed		[98]
Tennessee	2014	Tomato	Sporadic in the field		[99]
South Carolina	2017	Vegetables	Unusually severe and widespread		[100]

## Data Availability

Not applicable.
